# Feeding History Affects Intraguild Interactions between *Harmonia axyridis* (Coleoptera: Coccinellidae) and *Episyrphus balteatus* (Diptera: Syrphidae)

**DOI:** 10.1371/journal.pone.0128518

**Published:** 2015-06-01

**Authors:** Brecht Ingels, Pieter Van Hassel, Thomas Van Leeuwen, Patrick De Clercq

**Affiliations:** 1 Laboratory of Agrozoology, Department of Crop Protection, Faculty of Bioscience Engineering, Ghent University, Ghent, Belgium; 2 Institute for Biodiversity and Ecosystem Dynamics (IBED), University of Amsterdam (UvA), Amsterdam, the Netherlands; Federal University of Viçosa, BRAZIL

## Abstract

While the effect of several factors such as predator and prey size, morphology and developmental stage on intraguild predation (IGP) is widely investigated, little is known about the influence of diet on the occurrence and outcome of IGP. In the present study, the effect of the diet experienced during larval development on IGP between the ladybird *Harmonia axyridis* and the syrphid *Episyrphus balteatus* is investigated. Four diets were tested for *H*. *axyridis*: eggs of the Mediterranean flour moth *Ephestia kuehniella*, pea aphids, *Acyrthosiphon pisum*, in an *ad libitum* amount, pea aphids in a limited amount, and honey bee pollen. For *E*. *balteatus* only the two aphid diets were tested. First, experiments were performed to determine the quality of the various diets for development of both predators. Second, IGP experiments between *H*. *axyridis* and *E*. *balteatus* were performed both in Petri dishes and on potted pepper plants. The diet of both species influenced the incidence of IGP between *H*. *axyridis* and *E*. *balteatus* both in Petri dishes and on potted plants. In general, smaller larvae of *H*. *axyridis* (those fed on poor or restricted diet) fed more on hoverflies than large (well-nourished) ladybird larvae. Further, poorly nourished (smaller) larvae of *E*. *balteatus* were more susceptible to predation than well-fed (larger) hoverfly larvae. The observed effects were not only due to the lower fitness of larvae of both predators reared on an inferior quality diet but also to changes in predator behaviour. The results from this study show that IGP interactions are influenced by a multitude of factors, including feeding history of the organisms involved, and emphasize the importance of taking these factors into account in order to fully understand the ecological relevance of IGP.

## Introduction

Intraguild predation (IGP) is an interspecies interaction that has been widely investigated since the publication of Polis et al. [1]. IGP occurs when a predator consumes another predator which is competing for the same prey (i.e. a member of the same predatory guild) [1]. The aggressor is called the intraguild predator, the victim the intraguild prey, and their common resource the extraguild prey. It is a common and widespread interaction, that occurs in a variety of ecosystems and among species of numerous taxa (see [1] and [2] for a review). IGP is, for example, common among aphidophagous predators [3] and many other biological control agents [4].

As IGP is such a widespread interaction [2], it is important to understand the different mechanisms that influence its occurrence and outcome. The effects of various factors, such as predator and prey size, morphology and developmental stage, habitat complexity and the presence of extraguild prey on IGP have been thoroughly investigated [5,6,7]. However, much less is known about the influence of the previous diet and feeding regime experienced by intraguild predators and prey on their interactions. The few studies that have investigated diet-dependent aspects of IGP were mainly performed from a nutritional perspective [1,8,9]. Polis et al. [1] and Denno & Fagan [8] suggested that intraguild predators that were primarily feeding on nutrient-deficient food could compensate for the limitation of specific nutrients such as nitrogen through IGP. However, this hypothesis was not supported by the study of Kagata & Katyama [9], who investigated nitrogen content and nitrogen-use efficiency of the harlequin ladybird, *Harmonia axyridis* (Pallas) (Coleoptera: Coccinellidae), in intraguild interactions with the seven-spot ladybird, *Coccinella septempuncata* L. (Coleoptera: Coccinellidae).

Arguably, diet can affect the nutritional quality (i.e. the amount and proportion of nutrients) of the intraguild prey and/or physiological and behavioural traits like vigour and motility of both intraguild predator and prey, and as a consequence could affect the outcome of IGP [1,8,10]. For example, Yasuda *et al*. [11] showed that an increase in food supply resulted in a higher growth rate of the IG prey, leading to a lower incidence of IGP in interactions between ladybirds. Likewise, Ingels & De Clercq [7] observed that third instars of the hoverfly *Episyrphus balteatus* DeGeer (Diptera: Syrphidae) had higher activity and vigour in treatments where extraguild aphid prey were present, which could in part explain the lower frequency of IGP in confrontations with *H*. *axyridis* larvae.

In the present study, the potential effect of the previous diet experienced during larval development on intraguild interactions is further investigated, using IGP between larvae of *H*. *axyridis* and *E*. *balteatus*. *H*. *axyridis* is a predator of many aphid species and other soft-bodied arthropods [12,13] that is native to large parts of Asia, but has spread outside its native range. It has established in North and South America [13,14], Europe and some parts of Africa [15,16], where it is considered invasive (i.e. having direct ecological effects on native species, such as displacement of populations through direct predation or competition [17]). *E*. *balteatus* is the most common hoverfly in Europe, whose larvae are voracious predators of many aphid species [18,19]. It is an important biological control agent in various agro-ecosystems, especially in cereals [18,20]. This hoverfly has been observed to co-occur with the exotic coccinellid *H*. *axyridis* in various crops and in semi-natural habitats (see [21] and B. Ingels, personal observations).

The establishment of *H*. *axyridis* outside its native range has been associated with the decline of native aphid predators, in particular native coccinellids, in North America and Europe [22,23,24]. One of the mechanisms that has been hypothesized to contribute to the displacement of native natural enemies, is IGP. Laboratory studies have shown that *H*. *axyridis* is an intraguild predator of native ladybird species [5] and other aphid predators such as *E*. *balteatus* [7]. Furthermore, Hautier et al. [25], Thomas et al. [26], and Brown et al. [27] found evidence of IGP by *H*. *axyridis* in the field. The present study could further help to understand the mechanisms that influence IGP interactions between *H*. *axyridis* and native (non-coccinellid) aphid predators, and how IGP could contribute to the invasive success of *H*. *axyridis*.

Most, if not all, laboratory experiments on IGP involving *H*. *axyridis* were done with IG predators and prey that were reared in optimal conditions throughout their lifetime. Only during a short time period before the start of the experiments, the individuals used were starved in order to maximize the possibility that an IGP event would occur. In the present study, we set out to assess IGP interactions under conditions of food deprivation for a longer period of time. Our hypothesis is that a food restriction or a nutritionally inferior diet experienced during development will lead to larvae with lower fitness, resulting in IG predators that are less able to overcome the defences of their prey or in IG prey that are more vulnerable to IGP. First, experiments were conducted to compare the quality of the different food sources for supporting larval development of both predators. In these experiments, melanic and non-melanic individuals of *H*. *axyridis* were compared, as it has been shown that they can respond differentially to diet [28,29]. Second, several combinations were tested between larvae of both predators reared on the different diets to investigate how their feeding history affects IGP incidence. Experiments were done both in Petri dishes and on potted pepper plants, in order to assess the influence of spatial complexity on intraguild interactions.

## Materials and Methods

### Insects

In April 2011, individuals from an established wild population of *H*. *axyridis* were collected in a public park in Ghent, Belgium (Groene Vallei, 51°03’19”N 3°42’16”E). No specific permission was required to collect ladybirds from this location. From the collected ladybirds, two laboratory populations were initiated. The first population consisted of non-melanic *succinea* individuals, and will hence be referred to as the ‘non-melanic population’. Melanic *spectabilis* and *conspicua* individuals were used to start a second population, called the ‘melanic population’ [30]. Both populations were reared on frozen *E*. *kuehniella* eggs, as described by De Clercq et al. [31]. Eggs of *E*. *kuehniella* were obtained from Koppert BV (Berkel en Rodenrijs, The Netherlands).

A culture of *E*. *balteatus* was established with individuals collected in July 2011 in wheat fields near the Walloon Agricultural Research Centre in Gembloux, Belgium (50°34’08”N 4°43’25”E). Permission to collect syrphids on this private land was obtained from its owner, the Walloon Agricultural Research Centre (CRA-W). Adults were kept in Plexiglas cages (60 x 60 x 60 cm) and provided with pollen and honey water. Broad bean plants (*Vicia faba* L.) infested with the pea aphid *A*. *pisum*, were placed in the cages to allow syrphid oviposition. Emerged larvae were individually transferred to small Petri dishes (5 cm in diameter, 1.5 cm high) and fed *ad libitum* with pea aphids. All colonies were maintained at 23 ± 1°C, 65 ± 5% RH and a 16:8h (L:D) photoperiod.

### Diet quality experiments

To compare the quality of the different diets for *H*. *axyridis* and *E*. *balteatus*, experiments were performed to quantify the developmental time, larval weight, survival time under starvation and predation potential of larvae of both species that were reared on these diets. Four food sources were tested for *H*. *axyridis*: (i) eggs of the Mediterranean flour moth *Ephestia kuehniella* (Zeller) (Lepidoptera: Pyralidae) as a factitious food of high quality for *H*. *axyridis* [28,29], (ii) an *ad libitum* supply of the pea aphid *Acyrthosiphon pisum* (Harris) (Hemiptera: Aphididae) as a natural food for both *E*. *balteatus* and *H*. *axyridis*, (iii) a limited supply of *A*. *pisum* aphids, and (iv) honey bee pollen as a nutritionally inferior alternative food that does allow complete development of *H*. *axyridis* [28]. For *E*. *balteatus*, which is more restricted in its diet, only a limited and an *ad libitum* supply of aphids were investigated as feeding regimes. All experiments were conducted in a growth chamber at 23 ± 1°C, 65 ± 5% RH and a 16:8h (L:D) photoperiod. All experiments (including the IGP experiments described below) were carried out between October 2011 and May 2012.

#### Developmental time and larval weight

For *H*. *axyridis*, first instars of less than 24h old were individually transferred to a Petri dish (9 cm in diameter, 1.5 cm high) lined with a filter paper. A small piece of moistened household paper served as a water source. Depending on the treatment, one of the following food sources was added to the Petri dishes: (i) frozen eggs of *E*. *kuehniella* (*ad libitum*), (ii) an *ad libitum* number of pea aphids (*A*. *pisum*) or (iii) a limited number of pea aphids or (iv) frozen moist honey bee pollen (*ad libitum*). The honey bee pollen were obtained from Koppert BV (Berkel en Rodenrijs, The Netherlands). To provide an *ad libitum* supply of aphids, 10, 20, 30 or 40 aphids (a mixture of third and fourth instars) were supplied daily to individual first, second, third and fourth instars of the ladybird, respectively. In the treatment with a limited supply of aphids, larvae of *H*. *axyridis* received a daily amount of 1, 2, 3 or 4 aphids in the respective instars. Each treatment had 40 replicates, with 20 larvae from the melanic population and 20 from the non-melanic population. Each day, food was replenished and development and survival of the predator were checked. At least once a week, the larvae were transferred to a new Petri dishes with a clean filter paper. Maximum 24h after moulting to the fourth instar, the larvae of *H*. *axyridis* were weighed on a Sartorius Genius ME 215 P balance (± 0.01 mg). The experiment was terminated at adult emergence.

In the case of *E*. *balteatus*, first instars of maximum 24h old were individually transferred to an empty Petri dish (5 cm in diameter, 1.5 cm high). Because preliminary experiments indicated that there was no development on honey bee pollen and mortality was very high on *E*. *kuehniella* eggs, only two treatments were done: (i) an *ad libitum* supply of *A*. *pisum* or (ii) a limited supply of *A*. *pisum*. For an *ad libitum* supply of prey, 10, 20 or 30 aphids (a mixture of third and fourth instars) were presented to individual first, second and third instars of *E*. *balteatus*, respectively. In the treatment with a limited supply of aphids 2, 4 or 6 aphids were offered daily to individuals of the respective instars. There were 40 replicates per treatment. Food was replenished and development and survival were monitored on a daily basis. Maximum 24h after moulting to the third instar, the larvae of *E*. *balteatus* were weighed on a Sartorius Genius ME 215 P balance (± 0.01mg). The experiment was terminated at adult emergence.

#### Survival time under starvation

A second experiment assessed survival of the larvae of both predators without food, after having been reared on the diets described for each predator above. For both predators, 40 third instars were used for the experiments. For *H*. *axyridis*, only larvae from the non-melanic population were used, as there was no difference in developmental time and weight between both populations in the first experiment, and given that the non-melanic morph is the most abundant in Europe [15]. Maximum 24h after the larvae had reached the third instar on each of the tested diets, they were transferred to an individual Petri dish (9 cm in diameter in the case of *H*. *axyridis* and 5 cm in diameter in the case of *E*. *balteatus*) with only a moistened piece of household paper. The larvae were left in the dishes and checked every 24h for survival, until they died.

#### Predation capacity

A third experiment investigated the predation capacity of third instar larvae of both predators fed with the different diets. Again, for both predators and for each diet 40 larvae were reared up to the third instar as described above. For *H*. *axyridis*, only non-melanic individuals were used.

Newly moulted (<24h) third instars were transferred to individual Petri dishes (9 cm in diameter for *H*. *axyridis* and 5 cm in diameter for *E*. *balteatus*) containing 20 pea aphids (a mixture of third and fourth instars). After 6h, the remaining number of living aphids was counted. Given the short duration of the experiments, no plant material was added to the Petri dishes. A control treatment consisted of 30 petri dishes containing 20 aphids without a predator. To account for natural death of the aphids during the experiment, mortality was corrected using the formula of Abbott [32]:

$$\%\;\text{mortality}\;\text{by}\;\text{predation}=\left(\frac{\%\;\text{mortality}\;\text{in}\;\text{treatment}-\%\;\text{mortality}\;\text{in}\;\text{control}}{100-\%\;\text{mortality}\;\text{in}\;\text{control}}\right)\times 100$$

### IGP experiments

#### Petri dishes

To examine the influence of the food received during larval development on intraguild interactions between *H*. *axyridis* and *E*. *balteatus*, an IGP experiment in Petri dishes (5 cm in diameter) was conducted. IGP was investigated between third or fourth instars of *H*. *axyridis* on the one hand and third instars of *E*. *balteatus* on the other. The experiments by Ingels & De Clercq [7] indicated that third and fourth instars of *H*. *axyridis* acted most often as the intraguild predator in interactions with *E*. *balteatus*. Further, third instars of the hoverfly were more able to withstand attacks by the ladybird compared to younger larvae. It was thus assumed that a possible effect of larval diet on IGP would be most pronounced when these stages were combined.

Larvae of both predators were reared following the same methods as in the diet quality experiments described above. Newly moulted (<24h) individuals of one predator were confined in 5-cm Petri dishes together with one individual of the other predator. There was no additional starvation period. All possible combinations were made with larvae that had been fed on the different diets. As there were four tested diets for *H*. *axyridis*, and two for *E*. *balteatus*, 8 different combinations were tested for both third and fourth instars of the ladybird, resulting in 16 different combinations in total. Each combination was replicated 20 times.

Each experiment ran for 24h. During the first 90 minutes, the Petri dishes were observed in order to record all contacts among the predators. A contact was defined as a case where the predator larvae met by chance. Such a contact was registered as ‘without consequences’ if the predators separated again without any further action. A contact was registered as an attack when one predator tried to feed on the other. An attack was considered successful when it resulted in the death of the intraguild prey. After 24h, the survival of both insects was recorded.

#### Potted plants

In order to study the effects of larval food on IGP in larger arenas, the previous experiment was repeated using potted bell pepper plants. In this experiment, only third instars of *H*. *axyridis* were used, as the Petri dish experiments showed that the effect of larval diet was the strongest in combinations with this instar of the ladybird. Further, combinations with *H*. *axyridis* larvae fed on *E*. *kuehniella* eggs were not included. This resulted in a total of 6 tested combinations.

Larvae of both predators were reared on the different diets as described above. Newly moulted third instars of each predator species were transferred to the experimental arenas. The arenas consisted of a bell pepper plant (*C*. *annuum* cv. California Wonder) of approximately 15 cm high, placed in a plastic pot with a diameter of 20 cm. A transparent Plexiglas cylinder (18 cm in diameter, 20 cm high) was placed over the pepper plant, and pushed about 1 cm into the soil. The top of the cylinder was sealed with a mesh screen cloth. One individual of each species was placed in each arena. The larvae of *E*. *balteatus* were placed on one of the top leaves of the plant, while the *H*. *axyridis* larvae were placed at the base of the stem. After 2, 4, 6, 8 and 24h, the arenas were checked and the location and survival of the larvae was noted.

### Statistical analysis

Data analysis was carried out using SPSS 19.0 [33]. Count data (from the experiments on larval development and survival time under starvation) were analyzed using a generalized linear model with a Poisson error distribution and a log link function. Analysis of binomial data (from the IGP experiments) was also done with a generalized linear model, with in this case a binomial error distribution and a logit link function [34]. In both cases, analysis started each time with a saturated model and interactions and non-significant main factors were dropped at a significance level of 0.05. The results from the most parsimonious model are reported, using likelihood ratios to assure model fit. Weights and predation data followed a normal distribution, but did not have equal variances between groups. Thus, means were compared using an independent samples t-test or a one-way ANOVA with a Tamhane post-hoc test [35].

## Results

### Diet quality experiments with *H*. *axyridis*

#### Developmental time and larval weight

In the experiment on larval development of *H*. *axyridis* as a function of diet, there was no interaction between colour morph and diet (χ^2^ = 1.11; df = 4; P = 0.893) (Table 1). Furthermore, there was no influence of the colour morph on larval development (χ^2^ = 0.38; df = 1; P = 0.536). Consequently, the results for the two colour morphs were pooled for further analysis. Diet had a significant effect on developmental time (χ^2^ = 144.31; df = 4; P ≤ 0.001). There was no difference in developmental time between larvae fed with *E*. *kuehniella* or an *ad libitum* number of aphids (χ^2^ = 0.58; df = 1; P = 0.446) on the one hand, and between larvae fed with a limited number of aphids or pollen (χ^2^ = 1.27; df = 1; P = 0.260) on the other. However, the first two groups had a significantly shorter developmental time than the last two (χ^2^ ≥ 10.08; df = 1; P ≤ 0.002 for all contrasts). The longer development for *H*. *axyridis* larvae fed with pollen was caused by a slower development of all instars compared to larvae fed with a diet of *E*. *kuehniella* eggs or an *ad libitum* number of aphids. In contrast, for larvae supplied with a limited number of aphids, the fourth instar was characterized by a much longer duration compared to the other diets. Survival rates differed among treatments along the same lines as developmental times, with 97.5% and 80.0% survival for the treatments with *E*. *kuehniella* eggs and *ad libitum* aphids, and 55.0% and 57.5% survival on a limited supply of aphids and pollen, respectively.

**Table 1:** Total developmental time of *Harmonia axyridis* immatures (from first instar to adulthood), weight of the fourth instar, survival time under starvation of third instars and 6h predation rate by third instars (% of *Acyrthosiphon pisum* aphids killed) when offered various diets.

Diet	Developmental time (days)		Weight (mg)		Survival time under starvation (days)		Predation capacity (%)	
	n	mean ± SE	n	mean ± SE	n	mean ± SE	n	mean ± SE
***E*. *kuehniella***	39	16.72 ± 0.11 a	40	18.75 ± 1.08 a	40	7.63 ± 0.21 a	39	30.80 ± 2.43 ab
**Aphids ad lib**	32	17.47 ± 0.14 a	34	15.96 ± 0.70 a	35	4.11 ± 0.10 b	37	37.82 ± 2.54 a
**Aphids limited**	22	26.59 ± 0.44 b	24	9.26 ± 0.24 b	32	2.81 ± 0.07 c	35	29.12 ± 1.66 b
**Pollen**	24	28.33 ± 0.57 b	34	8.78 ± 0.36 b	37	2.95 ± 0.09 c	26	11.86 ± 1.49 c

Values in the same column followed by a different letter are significantly different. n: sample size.

Larval diet also affected the weight of fourth instar *H*. *axyridis* (F = 39.38; df = 4, 165; P ≤ 0.001) (Table 1). As for developmental duration, there was no difference between larvae given *E*. *kuehniella* or an *ad libitum* number of aphids (P = 0.297) and between those fed with a limited number of aphids or pollen (P = 0.960). However, larvae from the former two groups were significantly heavier than those from the latter two (P ≤ 0.001).

#### Survival time under starvation

The survival time under starvation of third instar *H*. *axyridis* was also influenced by the food received before the starvation period (χ^2^ = 119.97; df = 3; P ≤ 0.001) (Table 1). *E*. *kuehniella* eggs provided the highest energy reserves and consequently yielded a longer survival than the other food sources (χ^2^ ≥ 37.23; df = 1; P ≤ 0.001 for all contrasts). Furthermore, 92.5% of all third instars succeeded in reaching the fourth stadium in the latter treatment group, but then died as fourth instars. When aphids had previously been supplied in *ad libitum* numbers, survival of starved ladybird larvae was shorter than on *E*. *kuehniella* eggs, but longer than on the other diets (χ^2^ ≥ 6.92; df = 1; P ≤ 0.009 for both contrasts). On the *ad libitum* aphid diet, 45.7% of the third instar larvae successfully moulted. There was no difference in survival between ladybirds offered a limited number of aphids or pollen as food (χ^2^ = 0.10; df = 1; P = 0.745); no larvae reached the fourth instar in the latter treatments.

#### Predation capacity

The predation capacity of *H*. *axyridis* third instars also depended on their feeding history (F = 21.28; df = 3, 133; P ≤ 0.001). The number of aphids killed was the highest for larvae that had been reared in the previous instars on an *ad libitum* number of aphids, but this difference was only significant when compared to those that had been fed with a limited number of aphids or pollen (P ≤ 0.034 for both contrasts). The predation capacity of larvae reared on *E*. *kuehniella* eggs was similar to that of larvae in both aphid treatments (P ≥ 0.265). Larvae reared on pollen had the lowest predation capacity, being about one third of that in the other treatments (P ≤ 0.001 for all contrasts).

### Diet quality experiments with *E*. *balteatus*

#### Developmental time and larval weight

Diet affected developmental time of *E*. *balteatus* larvae (χ^2^ = 10.69; df = 1; P = 0.001), with a faster development when aphids were supplied *ad libitum* (Table 2). The longer development for larvae fed with a limited number of aphids was due to a prolongation of the third instar. Immature survival was also higher in the treatment with *ad libitum* aphids (52.5% compared to 32.5%). Further, the mean weight of larvae fed with an *ad libitum* number of aphids was about three times higher than that of larvae fed with a limited number of aphids (t = 9.847; df = 34; P ≤ 0.001).

**Table 2:** Total developmental time of *Episyrphus balteatus* immatures (from first instar to adulthood), weight of the third instar, survival time under starvation of third instars and 6h predation rate by third instars (% of *Acyrthosiphon pisum* aphids killed) when offered various diets.

Diet	Developmental time (days)		Weight (mg)		Survival time under starvation (days)		Predation capacity (%)	
	n	Mean ± SE	n	Mean ± SE	n	Mean ± SE	n	Mean ± SE
**Aphids ad lib**	21	13.29 ± 0.21 a	34	15.62 ± 1.10 a	29	4.59 ± 0.21 a	33	55.57 ± 4.40 a
**Aphids limited**	13	17.77 ± 0.58 b	32	4.65 ± 0.14 b	28	2.89 ± 0.17 b	28	58.90 ± 4.30 a

Values in the same column followed by a different letter are significantly different. n: sample size.

#### Predation capacity and survival time under starvation

The survival of third instars of *E*. *balteatus* in the absence of prey was also influenced by feeding history (χ^2^ = 10.69; df = 1; P = 0.001), with a longer survival for larvae that had been supplied with an *ad libitum* number of aphids. None of the starved larvae succeeded in reaching the pupal stage. The predation capacity of third instar hoverfly larvae, however, was independent of their feeding history (t = -0.536; df = 59; P = 0.594).

### IGP experiments

#### Petri dishes

The mean number of contacts (sum of the contacts that led to an attack and those without consequences) and success rate of attacks observed during the first 90 minutes of the experiments is shown in Table 3. In general, the number of contacts was higher when the *H*. *axyridis* larvae were reared on a limited aphid supply or on bee pollen. In about 30 to 50% of cases, a contact led to an attack by *H*. *axyridis*. In general, the coccinellid was the most aggressive, and events noted as attacks by *E*. *balteatus* were mostly counterattacks. An attack by *E*. *balteatus* was never successful. In contrast, the success rate of an attack by *H*. *axyridis* varied between 0 and 25%. For both larval instars of the ladybird, there were more successful attacks on hoverfly larvae with a limited aphid supply compared to those given aphids *ad libitum*. Furthermore, the number of successful attacks was higher for fourth instars than for third instars of *H*. *axyridis*.

**Table 3:** Number of contacts (sum of the contacts that led to an attack and those without consequences) during the first 90 min of the Petri dish IGP experiments, number of contacts that resulted in an attack by *Harmonia axyridis* or *Episyrphus balteatus* and total number of replicates in which the attack by *H*. *axyridis* was successful (n = 20) for each combination of larvae reared on the various diets (means ± SE). L3–L4: third and fourth instar of *H*. *axyridis*.

Diet *H*. *axyridis*		Diet *E*. *balteatus*	No. of contacts	No. of attacks by *H*. *axyridis*	No. of attacks by *E*. *balteatus*	Replicates with successful attack by *H*. *axyridis*
L3	*E*. *kuehniella*	Aphids ad lib	1.90 ± 0.49	0.70 ± 0.27	0.40 ± 0.13	0
		Aphids limited	4.05 ± 1.25	1.80 ± 0.54	0.60 ± 0.28	1
	Aphids ad lib	Aphids ad lib	3.10 ± 0.91	0.70 ±0.19	0.25 ± 0.12	0
		Aphids limited	3.90 ± 1.18	1.20 ± 0.37	0.85 ± 0.40	1
	Aphids limited	Aphids ad lib	9.05 ± 1.99	4.65 ± 1.06	0.65 ± 0.18	1
		Aphids limited	7.05 ± 1.22	3.20 ± 0.66	0.95 ± 0.44	9
	Pollen	Aphids ad lib	3.20 ± 0.95	1.00 ± 0.41	0.65 ± 0.25	0
		Aphids limited	4.65 ± 1.05	1.55 ± 0.47	0.85 ± 0.27	1
L4	*E*. *kuehniella*	Aphids ad lib	3.00 ± 0.73	1.70 ± 0.43	0.40 ± 0.15	3
		Aphids limited	1.90 ± 0.70	0.65 ± 0.30	0.25 ± 0.12	3
	Aphids ad lib	Aphids ad lib	3.60 ± 1.45	0.35 ± 0.22	0.45 ± 0.22	1
		Aphids limited	4.75 ± 1.18	1.60 ± 0.47	1.05 ± 0.48	3
	Aphids limited	Aphids ad lib	9.70 ± 2.42	4.20 ± 0.97	0.35 ± 0.17	8
		Aphids limited	4.60 ± 0.92	2.00 ± 0.35	0.65 ± 0.36	10
	Pollen	Aphids ad lib	6.05 ± 1.23	2.70 ± 0.59	0.65 ± 0.21	6
		Aphids limited	2.80 ± 0.87	1.00 ± 0.31	0.30 ± 0.18	5

**Fig 1:**
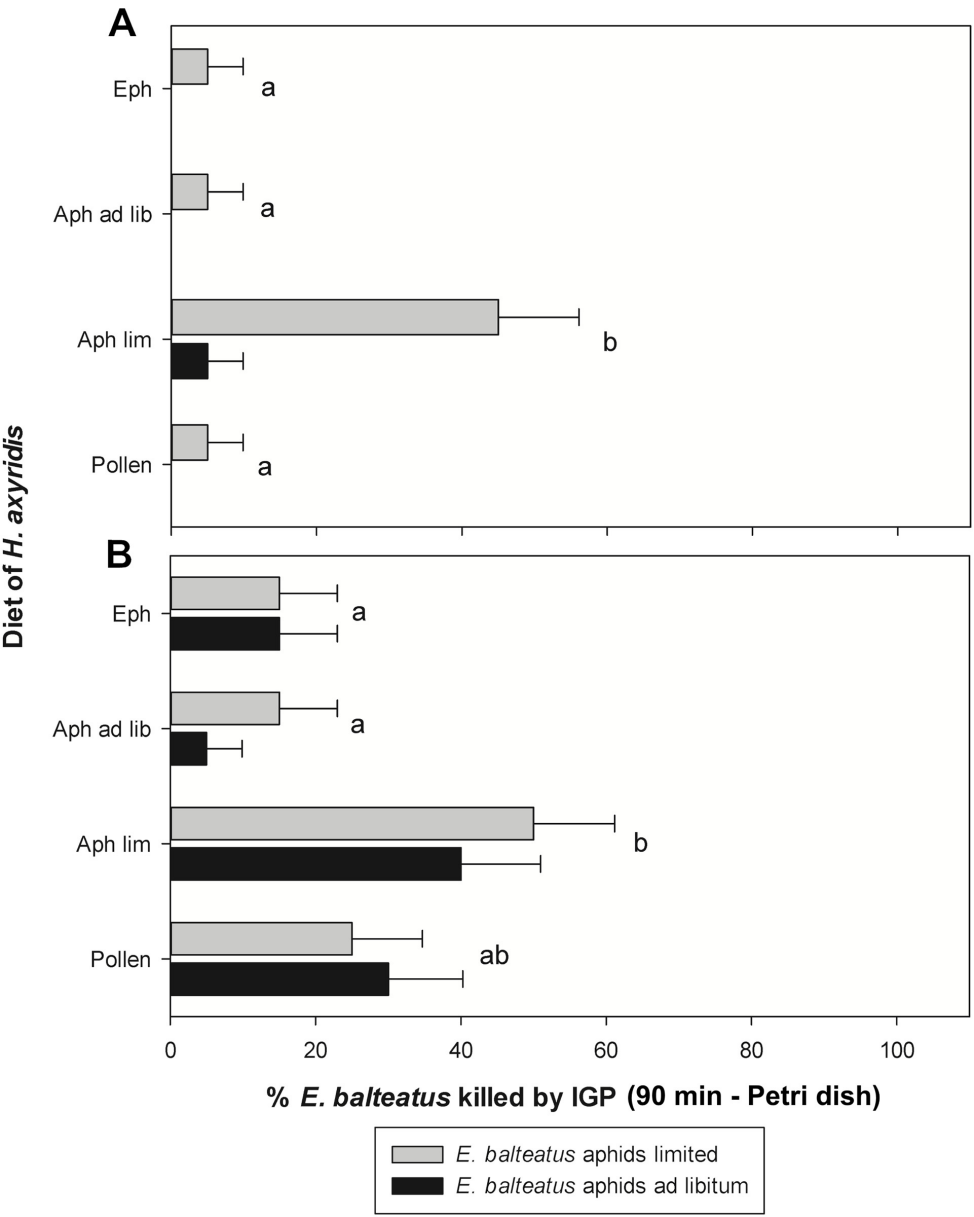
IGP by *Harmonia axyridis* on *Episyrphus balteatus* in Petri dishes after 90 minutes. Percentage of third instars of *Episyrphus balteatus* reared on an *ad libitum* or limited supply of aphids that were killed through IGP in Petri dishes after 90 minutes by third (A) and fourth (B) instars of *Harmonia axyridis* reared on various diets. Eph: *Ephestia kuehniella* eggs, Aph ad lib: *ad libitum* supply of *Acyrthosiphon pisum* aphids, Aph lim: limited supply of aphids, Pollen: moist honey bee pollen. The effect of larval diet of *H*. *axyridis* is indicated by lowercase letters (generalized linear model with Poisson error distribution and a log link function); within each panel, groups of bars followed by a different letter are significantly different. Error bars represent SE-values.

The incidence of IGP by third and fourth instars of *H*. *axyridis* on *E*. *balteatus* after 90 minutes is shown in Fig 1. There was a significant interaction between the diet of *E*. *balteatus* and the tested stage of *H*. *axyridis* (χ^2^ = 4.92; df = 1; P = 0.026). For third instars of *H*. *axyridis*, there was a significant effect of previous diet of *E*. *balteatus* on the incidence of IGP (χ^2^ = 7.34; df = 1; P = 0.007). More hoverfly larvae were killed during the first 90 minutes when they had been reared on a limited aphid supply versus an *ad libitum* aphid supply, irrespective of the larval feeding regime of *H*. *axyridis*. Further, the larval diet of *H*. *axyridis* also affected the incidence of IGP (χ^2^ = 15.09; df = 3; P = 0.002). When *H*. *axyridis* larvae had received a limited number of aphids during their development, a higher number of *E*. *balteatus* larvae was killed than in all other treatments (χ^2^ = 6.52; df = 1; P = 0.011 for all contrasts).

In the combinations with fourth instars of *H*. *axyridis*, the diet of *E*. *balteatus* did not affect the occurrence of IGP (χ^2^ = 0.34; df = 1; P = 0.561). In contrast, there was again a significant effect of the feeding history of the *H*. *axyridis* larvae (χ^2^ = 14.27; df = 3; P = 0.003). IGP on *E*. *balteatus* occurred more frequently when *H*. *axyridis* had been given a limited aphid supply compared to the treatments with *E*. *kuehniella* eggs or an *ad libitum* supply of aphids (χ^2^ ≥ 7.94; df = 1; P ≤ 0.005 for both contrasts). For the ladybird larvae that had been reared on pollen, there was an intermediate level of IGP, that was not significantly different from any other treatment (χ^2^ ≤ 2.617; df = 1; P ≥ 0.106).

**Fig 2:**
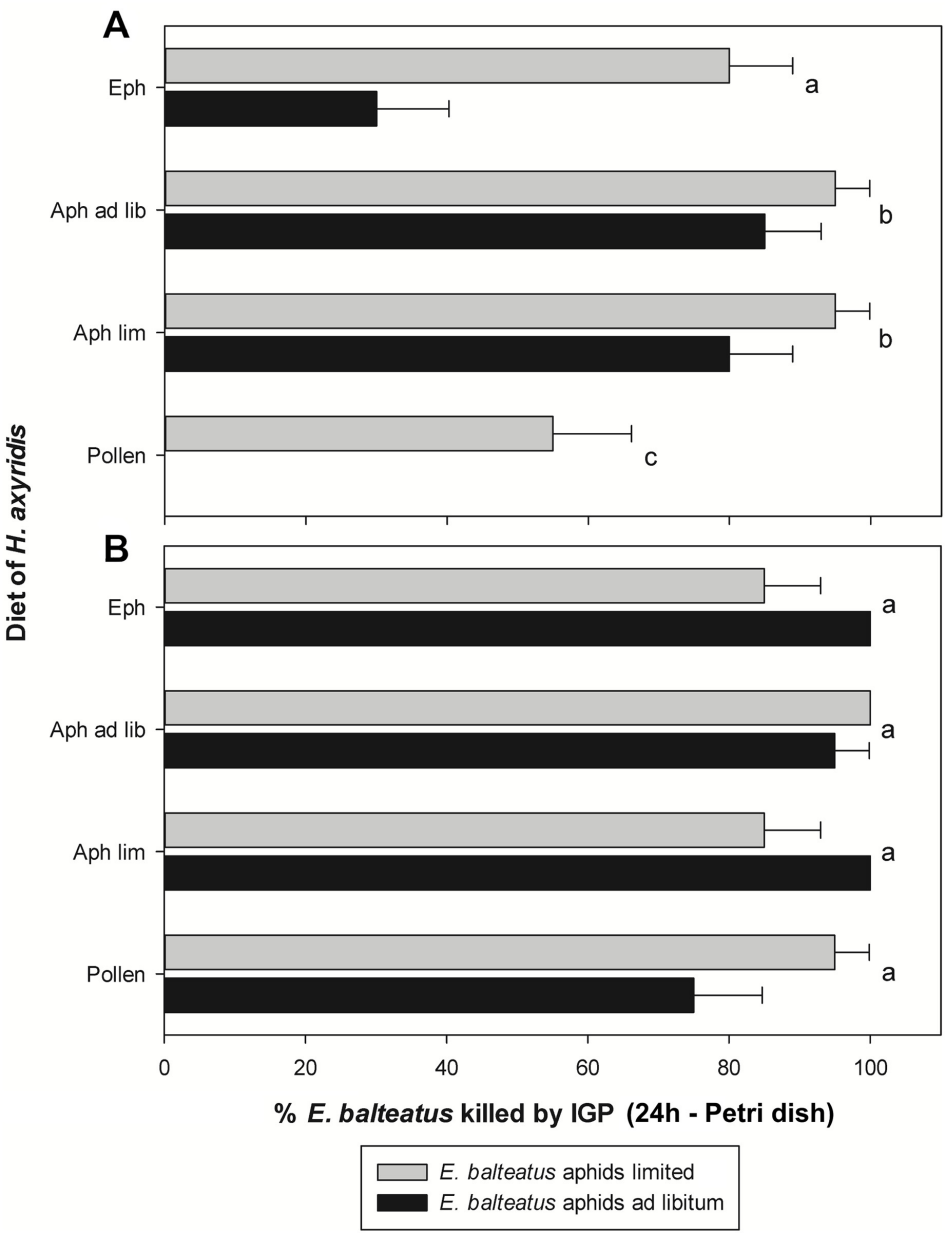
IGP by *Harmonia axyridis* on *Episyrphus balteatus* in Petri dishes after 24h. Percentage of third instars of *Episyrphus balteatus* reared on an *ad libitum* or limited supply of aphids that were killed through IGP in Petri dishes after 24h by third (A) and fourth (B) instars of *Harmonia axyridis* reared on various diets. Eph: *Ephestia kuehniella* eggs, Aph ad lib: *ad libitum* supply of *Acyrthosiphon pisum* aphids, Aph lim: limited supply of aphids, Pollen: moist honey bee pollen. The effect of larval diet of *H*. *axyridis* is indicated by lowercase letters (generalized linear model with Poisson error distribution and a log link function); within each panel, groups of bars followed by a different letter are significantly different. Error bars represent SE-values.

The occurrence of IGP by both instars of *H*. *axyridis* on *E*. *balteatus* after 24h is shown in Fig 2. Again, there was a significant interaction between the food source of *E*. *balteatus* and the developmental stage of *H*. *axyridis* (χ^2^ = 9.87; df = 1; P = 0.002). In the combinations with third instars of *H*. *axyridis*, IGP still occurred more often on *E*. *balteatus* larvae that had experienced a limited aphid supply compared to those fed aphids *ad libitum* (χ^2^ = 22.32; df = 1; P ≤ 0.001). The larval diet of *H*. *axyridis* also influenced the incidence of IGP (χ^2^ = 37.84; df = 1; P ≤ 0.001). The lowest incidence of IGP was noted when *H*. *axyridis* was fed on pollen (χ^2^ ≥ 7.82; df = 1; P ≤ 0.005 for all contrasts). When pollen-fed ladybird larvae were combined with *E*. *balteatus* larvae fed aphids *ad libitum*, all hoverfly larvae survived. In the treatment where *H*. *axyridis* received *E*. *kuehniella* eggs, IGP occurred more frequently than in the pollen group, but with a lower frequency than in both aphid treatments (χ^2^ ≥ 11.55; df = 1; P ≤ 0.001). No difference in the incidence of IGP was found between *H*. *axyridis* larvae fed aphids *ad libitum* or in limited numbers (χ^2^ = 0.59; df = 1; P = 0.441).

For fourth instars of *H*. *axyridis*, the incidence of IGP after 24h was independent of the diet of both *E*. *balteatus* and *H*. *axyridis* (χ^2^ = 0.09; df = 1; P = 0.769 and χ^2^ = 3.68; df = 1; P = 0.289, respectively). Thus, fourth instars of the ladybird could overcome third instars of the hoverfly, whatever food regime they had experienced during their development, in almost all confrontations.

It is worth noting that in the treatment with pollen 22.5% of the tested *H*. *axyridis* larvae (7 third instars and 2 fourth instars) died during the IGP experiment. These deaths were caused by weakness due to insufficient food and/or by defensive slime secretions from *E*. *balteatus* larvae. However, *E*. *balteatus* were never observed to feed on weakened or dead *H*. *axyridis* larvae.

#### Potted plants

**Fig 3:**
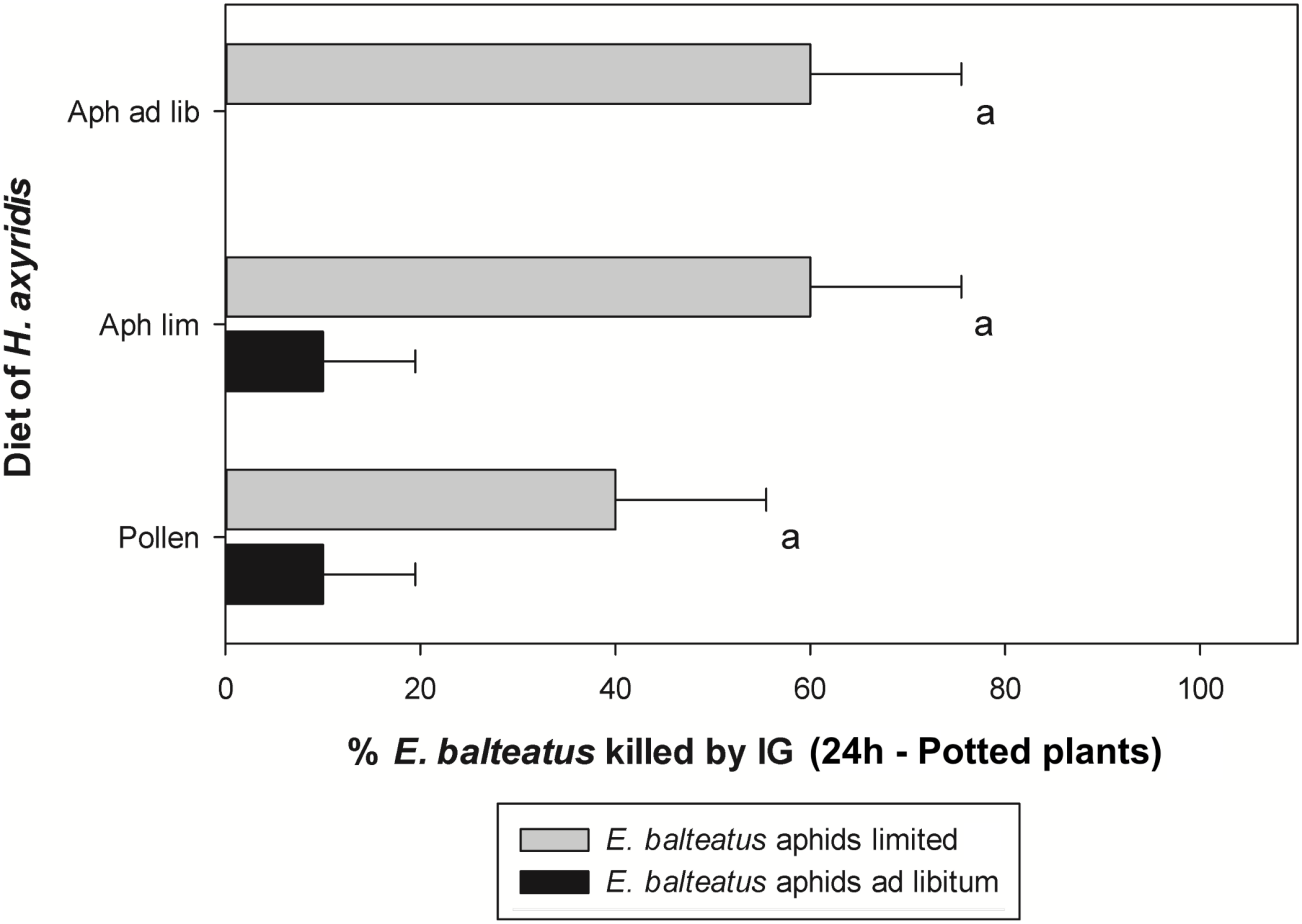
IGP by *Harmonia axyridis* on *Episyrphus balteatus* on potted pepper plants after 24h. Percentage of third instars of *Episyrphus balteatus* reared on an *ad libitum* or limited supply of aphids that were killed through IGP on pepper plants after 24h by third instars of *Harmonia axyridis* reared on various diets. Aph ad lib: *ad libitum* supply of *Acyrthosiphon pisum* aphids, Aph lim: limited supply of aphids, Pollen: moist honey bee pollen. No statistically significant effect of larval diet of *H*. *axyridis* was found (generalized linear model with Poisson error distribution and a log link function). Error bars represent SE-values.

For each observation time, there was no interaction between the diet of *H*. *axyridis* and that of *E*. *balteatus* (χ^2^ ≤ 0.22; df = 2; P ≥ 0.898 for all contrasts). Only after 24h, there was a significant effect of the previous diet of *E*. *balteatus* on the incidence of IGP (χ^2^ = 11.56; df = 1; P = 0.001) (Fig 3). Hoverfly larvae fed with an *ad libitum* number of aphids survived better than those given a limited aphid supply. No significant effect of the diet of *H*. *axyridis* was found (χ^2^ = 0.64; df = 2; P = 0.727).

When comparing the combinations that were performed in Petri dishes and on potted plants, a significant interaction between the diet of *H*. *axyridis* and the size of the arena was found (χ^2^ = 15.53; df = 2; P ≤ 0.001), indicating a different effect of the arena size depending on the food source of the ladybird. Therefore the data for each food source of *H*. *axyridis* were analyzed separately. IGP was less frequent on plants compared to Petri dishes for ladybird larvae reared on a limited and *ad libitum* supply of aphids (χ^2^ = 13.47; df = 1; P ≤ 0.001 and χ^2^ = 18.86; df = 1; P ≤ 0.001, respectively). For *H*. *axyridis* larvae fed with pollen, there was no influence of the arena size on the incidence of IGP (χ^2^ = 0.06; df = 1; P = 0.808).

## Discussion

Food ration and quality substantially affected the fitness of larvae of both *H*. *axyridis* and *E*. *balteatus*. For *H*. *axyridis*, the developmental time and weight of the fourth instar were similar for larvae with an *ad libitum* supply of *E*. *kuehniella* eggs (a factitious food) or pea aphids (a natural food), which is in agreement with the findings of Specty et al. [36] and Berkvens et al. [29]. Furthermore, there was no difference in the predation capacity of these groups. However, third instars of *H*. *axyridis* survived longer without food when they had been reared in previous instars on *E*. *kuehniella* eggs as compared to aphids *ad libitum*. Based on these results and the findings of Berkvens et al. [29], *E*. *kuehniella* eggs were classified as the highest quality diet for *H*. *axyridis* among the tested diets. Larvae of the ladybird reared on a limited number of aphids showed lower fitness than those offered the former two diets. Likewise, Dmitriew and Rowe [37,38] found that limiting the supply of pea aphids led to a slower development and smaller larvae of *H*. *axyridis*, compared to an *ad libitum* supply of aphids. Developmental parameters of *H*. *axyridis* larvae reared on moist honey bee pollen were similar to those of predators fed with a restricted number of aphids. However, as the predation capacity of the former group was lower, pollen were classified as the lowest quality diet tested here. For *E*. *balteatus*, all developmental, starvation and predation parameters were superior for larvae supplied with aphids *ad libitum* compared to those receiving a limited supply of aphids.

The variation in fitness between larvae with a different feeding history also led to differences in the incidence of IGP between *H*. *axyridis* and *E*. *balteatus*. Like in Ingels & De Clercq [7], IGP was asymmetric in favour of the coccinellid in all experiments in this study. After 90 min in the Petri dish experiments, there was a clear influence of previous diet of *H*. *axyridis* on the frequency of IGP. In combinations with third instars of the ladybird, IGP occurred more frequently when *H*. *axyridis* larvae had received a limited supply of aphids compared to all other treatments. This could be explained by a difference in profitability of further feeding between large and small larvae. Larger larvae, such as those from the treatments with *E*. *kuehniella* eggs or *ad libitum* aphids, may have less to gain (in terms of fitness) from further growth compared to smaller larvae for whom more food may be critical for survival. Ladybird larvae fed on a low number of aphids were smaller and had lower energy reserves, and were thus more likely to search for prey. This hypothesis is supported by the higher number of contacts registered between both predators in combinations with ladybird larvae offered limited numbers of aphids compared to those fed on the higher quality diets.

When *H*. *axyridis* larvae had been reared on pollen, a higher number of contacts was also observed. However, for third instars this did not result in a higher incidence of IGP. This could be explained by the low quality of pollen as a food for *H*. *axyridis*, so that the ladybird larvae were too weak to overcome the defences of the hoverfly larvae. In the combinations with fourth instars of *H*. *axyridis*, the effect of diet of the ladybird larvae was similar as in the third instar, with only the frequency of IGP being relatively higher for the pollen treatment.

After 24h in the experimental Petri dishes, the influence of the previous diet of *H*. *axyridis* on the frequency of IGP, as noted after 90 min, could no longer be observed in combinations with fourth instars of the ladybird. IGP occurred in 80 to 100% of cases, indicating that fourth instars of *H*. *axyridis* can easily overcome the defences of *E*. *balteatus* larvae, independent of their diet. In combinations with third instars of the ladybird, the effect of diet of *H*. *axyridis* was still present, but changed as compared to the situation after 90 min. First, IGP levels for both aphid diets were no longer different. Because of the small size of the Petri dish arenas, numerous contacts between both predators may have occurred during the course of the experiment, leading to a high number of opportunities for IGP to take place. That way, even the slowest of ladybird larvae could have met hoverfly larvae often enough to consume them, leading to no observable differences in IGP. Second, the incidence of IGP was lower for ladybirds reared on *E*. *kuehniella* eggs than for those reared on aphids. This was surprising, as the former group of coccinellid larvae had the highest fitness level, and consequently were expected to be equally able to kill a hoverfly larva as their counterparts fed with aphids. However, *H*. *axyridis* larvae reared on *E*. *kuehniella* eggs survived longer without food than those fed with aphids, indicating that they possessed higher energy reserves. Because of these higher energy reserves, *H*. *axyridis* larvae fed on *E*. *kuehniella* are likely to have less to gain from consuming additional prey as compared to ladybird larvae fed on aphid prey, which could have resulted in a lower tendency to attack *E*. *balteatus* larvae.

On potted plants, IGP levels showed the same trend as observed in the Petri dish experiments after 24h (i.e. similar levels of IGP for the two aphid diets, and a lower level of IGP for the pollen treatment). Despite a lack of significant differences in overall IGP incidence among the treatments, IGP occurred earlier for *H*. *axyridis* that had developed on a limited aphid supply than in the other two treatments. For example, whereas the first IGP events took place within 2h in the former case, it took up to 6h when *H*. *axyridis* was reared on *ad libitum* aphids. These observations corroborate the results from the Petri dish experiments that hungry predators search more actively for prey.

For the treatments with *H*. *axyridis* larvae reared on an *ad libitum* versus limited supply of aphids, the frequency of IGP on plants was significantly lower compared to the same combinations in Petri dishes. These results are not in line with the findings of Ingels & De Clercq [7], where IGP levels were not different between Petri dishes and potted broad bean plants for combinations with third instars of both predators. However, the plant arenas used in the present study were larger than those used by Ingels & De Clercq [7]. Hindayana et al. [6] also found a decrease in IGP incidence between *C*. *septempunctata* and *E*. *balteatus* when the arena size was increased from a Petri dish to a potted plant arena of similar size as the one used in the present study. Like in Ingels & De Clercq [7], the effect of habitat complexity was, however, not consistent in the present study, as there was no difference in the frequency of IGP between Petri dishes and potted plants for the treatment with *H*. *axyridis* larvae fed on pollen. Probably, ladybird larvae reared on pollen were too weak for the effect of the arena to be important. Lower IGP levels recorded in the plant arenas could be further explained by the differences in migration behaviour between *H*. *axyridis* and *E*. *balteatus*. As was observed in Ingels & De Clercq [7], *H*. *axyridis* larvae tended to migrate away from the plants and were consequently found more often on the Plexiglas cylinder or on the soil whereas *E*. *balteatus* remained more on the plant. This could have led to a lower number of encounters and thus to a lower IGP incidence.

The diet of *E*. *balteatus* also influenced the incidence of IGP, and this in all combinations with third instars of *H*. *axyridis* (in Petri dishes, both after 90 min and 24h, and on plants). In general, IGP occurred more frequently on hoverfly larvae that had developed on a limited supply of aphids than on those fed aphids *ad libitum*. The lower fitness of the former group of syrphid larvae may thus have affected their ability to withstand attacks of *H*. *axyridis*, making them more vulnerable to IGP. In the combinations with fourth instars of *H*. *axyridis*, however, there was no effect of the feeding history of *E*. *balteatus*. The frequency of IGP in these combinations was also higher than with third instars of *H*. *axyridis*, indicating again that fourth instars of the ladybird can easily overpower *E*. *balteatus* larvae, regardless of the syrphid's diet.

In the experiments by Lucas et al. [39] and Mendel & Schausberger [40] similar results as those found in the present study were reported for other arthropod predators. Lucas et al. [39] investigated the influence of different extraguild resources on the direction and intensity of IGP between the mirid bugs *Macrolophus caliginosus* Wagner (intraguild prey) and *Dicyphus tamaninii* Wagner (intraguild predator) (both Hemiptera: Miridae). They found that providing a food resource to the intraguild prey during the 5 days before the IGP experiment resulted in a significant decrease in the level of IGP compared to the treatment where the intraguild prey was starved. Furthermore, it was shown that the decrease in IGP intensity was stronger if the resources available to the intraguild prey were of higher quality (i.e., allowing a faster development). Like in the present study, the authors concluded that the level of satiation was an important driving force promoting IGP. Less satiated individuals were assumed to spend more time foraging, thus enhancing the encounter possibilities between intraguild prey and predator.

Mendel & Schausberger [40] found that IGP among the predatory mites *Neoseiulus californicus* McGregor and *Neoseiulus cucumeris* (Oudemans) (Acari: Phytoseiidae) was influenced by the diet of both intraguild predator and prey. Their findings were in line with our study, in that not only the outcome of IGP interactions was affected, but also the speed at which intraguild prey were attacked. For example, *N*. *californicus* females attacked their intraguild prey sooner when they were previously fed with the two-spotted spider mite *Tetranychus urticae* Koch (Acari: Tetranychidae) (considered a high quality food) compared to females fed with pollen (a lower quality food). This was explained by the fact that spider mite-fed predators were better nourished than pollen-fed predators, which positively affected their ability to pursue, capture and overwhelm their prey. The diet consumed by the intraguild prey also affected the attack latencies. Pollen-fed prey were earlier attacked and killed than spider mite-fed prey. The authors suggested several explanations for this. It could be that pollen had a lower nutritional value for the intraguild prey than spider mites, leading to a lower vigour and slower movements of pollen-fed individuals, which made them easier to capture by their intraguild predators. Alternatively or additionally, it was also assumed that, because of a higher hunger level, pollen-fed individuals could have been more actively searching for food, increasing the encounter rate with the predator and consequently shorten the attack latencies.

In conclusion, our results indicate that the outcome of IGP between *H*. *axyridis* and *E*. *balteatus* is affected by the feeding history of both predators. The effects may not only be the consequence of lower fitness and less effective defence strategies when food is limited in supply or of lower quality, but may also be related to changes in predator behaviour. Whether or not an IGP event will take place may depend on a trade-off between available energy reserves (and thus the necessity to search for and attack prey) on the one hand, and weakened defences (that result in a lower tendency to take the risk associated with attacking an intraguild prey) on the other. This points out that the outcome of intraguild interactions is influenced by a multitude of factors, including feeding history of the organisms involved, and emphasizes the importance of taking these factors into account in order to fully understand the ecological relevance of these interactions. Given the limitations of a laboratory setting, more field realistic research is needed to investigate intraguild interactions between *H*. *axyridis* and both coccinellid and non-coccinellid predators, and to their contribution to the invasive success of *H*. *axyridis*.
